# A phase II study in advanced cancer patients to evaluate the early transition to palliative care (the PREPArE trial): protocol study for a randomized controlled trial

**DOI:** 10.1186/s13063-015-0655-8

**Published:** 2015-04-12

**Authors:** Thamires Monteiro do Carmo, Bianca Sakamoto Ribeiro Paiva, Milena Ruas de Siqueira, Luciana de Toledo Bernardes da Rosa, Cleyton Zanardo de Oliveira, Maria Salete de Angelis Nascimento, Carlos Eduardo Paiva

**Affiliations:** Palliative Care and Health-Related Quality of Life Research Group (GPQual), Barretos Cancer Hospital, Pio XII Foundation, Barretos, São Paulo Brazil; Center for Research Support - NAP, Barretos Cancer Hospital, Pio XII Foundation, Barretos, São Paulo Brazil; Human Sciences Institute - Psychologist Course, Universidade Paulista (UNIP), São José do Rio Preto, São Paulo Brazil; Palliative Care Department, Barretos Cancer Hospital, Pio XII Foundation, Barretos, São Paulo Brazil; Department of Clinical Oncology, Barretos Cancer Hospital, Pio XII Foundation, Barretos, São Paulo Brazil; Departamento de Oncologia Clínica, Divisão de Mama e Ginecologia, Rua Antenor Duarte Vilella, Bairro Dr Paulo Prata, 1331 Barretos, SP Brazil

**Keywords:** depression, neoplasms, palliative care, cognitive behavioral therapy

## Abstract

**Background:**

Previous studies have demonstrated the benefit of early integration of palliative care (PC) in oncology. However, patients continue to receive late referrals to PC even in comprehensive cancer centers. Patients and health professionals may perceive PC as ‘a place to die,’ and this stigma is a barrier to timely referrals and to patient acceptance of treatment.

**Methods/design:**

The primary objective is to evaluate the feasibility of psychosocial intervention and PC in patients with advanced cancer. The patients will be submitted to a series of brief psychosocial interventions that are based on cognitive behavioral therapy, and patient acceptance and satisfaction will be assessed. In addition, the impact of these interventions on depressive symptoms will be evaluated. A randomized, open-label, phase II trial with two intervention arms and a control group will be conducted. Patients who are started on palliative chemotherapy and who meet the inclusion criteria will be enrolled. The study participants will be recruited from the outpatient oncology clinics at Barretos Cancer Hospital and will be randomized into one of the following three treatment arms: Arm A, which will include five weekly psychosocial interventions based on CBT in combination with early PC; Arm B, which will include early PC only; and Arm C, which will include standard oncologic care. The Hospital Anxiety and Depression Scale (HADS), the Patient Health Questionnaire (PHQ-9), the Edmonton Symptom Assessment System (ESAS-br), the Family Satisfaction with End-of-Life Care (FAMCARE)-Patient scale, and the Disease Understanding Protocol will be used for data collection. The patients will answer these questionnaires at baseline and 45, 90, 120 and 180 days after randomization.

**Discussion:**

Despite evidence of the positive impact of early PC, it is often provided to patients only at later stages. The inadequate awareness and stigmatization of PC as a place to die are barriers that complicate the early referral. Patients with advanced cancer may benefit from a psychosocial and educational strategy that adequately prepares them for initial PC appointments after an early referral to PC. We anticipate that benefits of psychological intervention shall be synergistic to secondary emotional benefits from the early integration of PC.

**Trial registration:**

This trial was registered on 6 May 2014 with ClinicalTrials.gov (identifier: NCT02133274).

**Electronic supplementary material:**

The online version of this article (doi:10.1186/s13063-015-0655-8) contains supplementary material, which is available to authorized users.

## Background

Approximately 32.6 million people worldwide are living with cancer that has been diagnosed within the last five years [1]. Many cancer cases are diagnosed as metastatic disease despite advances in cancer prevention strategies. Furthermore, a significant portion of these cases are associated with disease recurrence during the monitoring period. These advanced disease cases are eligible for concurrent or exclusive monitoring by specialized palliative care (PC) teams. Approximately 6.5 million cancer patients are in PC worldwide [2].

The World Health Organization has defined PC as ‘an approach that improves the quality of life (QL) of patients and families who face a life-threatening illness through the prevention and relief of suffering. This approach is based on the early identification, assessment, and treatment of pain and other physical, psychosocial, and spiritual problems’ [3]. Therefore, PC should be provided by a multidisciplinary team that acts integrally in outpatient, hospital, and/or home environments [4,5].

Temel *et al*. [6] evaluated the early integration of PC in non-small cell lung cancer patients who were starting first-line palliative chemotherapy. An improvement in the QL scores and a decrease in the symptoms of depression were evident 12 weeks after randomization. Furthermore, they found that the patients who received early PC were submitted to fewer aggressive treatments in the final stages of life. A post-hoc analysis inclusively demonstrated a 2.7-month increase in the overall survival rate of patients who received PC. A study by Bakitas *et al*. [7] evaluated the early introduction of a psychoeducational program in the treatment of cancer patients from rural areas in the United States. The authors observed improved QL scores and reduced symptoms of depression over time. More recently, a cluster randomized clinical trial [8] evaluated the early introduction of PC in a general oncology population in Canada. The main objective of this study was to improve the spiritual QL of patients 3 months after randomization. Improvements in the overall QL at 3 and 4 months after randomization, the spiritual QL after 4 months and patient satisfaction were observed in the patients who received early PC.

Despite evidence of the positive impact of early PC on the progress of cancer patients, PC is often provided to patients at later stages even at oncology and PC reference centers [9]. In Brazil, reliable information on how and when cancer patients are referred to PC is lacking; however, we believe that patients are referred late to PC. The inadequate awareness and stigmatization of PC, which is perceived as a place to die, are barriers that complicate the early referral of patients to PC [10,11]. The title ‘Palliative Care’, which implies improvisation or something provisional, has complicated patient acceptance of PC within the context of early cancer support during chemotherapy. Patients who are referred to PC are often anxious at the first appointment [12], which leads to a high absenteeism rate.

Therefore, the main hypothesis of the present study is that patients with advanced cancer may benefit from a psychosocial and educational strategy that adequately prepares them for initial PC appointments after an early referral to PC.

## Methods/design

### Main objectives

The main objectives of this study are as follows:

1. To assess the feasibility of a brief psychosocial intervention based on cognitive behavioral therapy (CBT) and early PC in advanced cancer patients, including patient acceptance and satisfaction.
2. To assess the impact of the intervention on symptoms of depression at 90 days after randomization and to compare the differences between the intervention groups and the control group and between the intervention groups.

### Secondary objectives

The secondary objectives of the study are as follows:

1. To assess the impact of the interventions on symptoms of depression at 45, 120 and 180 days after randomization and to compare the differences between the intervention groups and the control group and between the intervention groups.
2. To assess the impact of the interventions on anxiety symptoms at 45, 90, 120 and 180 days after randomization and to compare the differences between the intervention groups and the control group and between the intervention groups.
3. To assess the impact of the interventions on the QL scores at 45, 90, 120 and 180 days after randomization and to compare the differences between the intervention groups and the control group and between the intervention groups;
4. To assess the impact of the interventions on symptom scores (pain, fatigue, nausea, drowsiness, anorexia, dyspnea, and insomnia) at 45, 90, 120 and 180 days after randomization and to compare the differences between the intervention groups and the control group and between the intervention groups.
5. To assess the differences in disease awareness at 45, 90, 120 and 180 days after randomization in the three arms of the trial.
6. To assess the differences in the number of invasive procedures at the end of life, such as intensive care unit admissions and chemotherapy during the last 14 to 28 days of life, between the treatment arms.

### Study design

**Figure 1:**
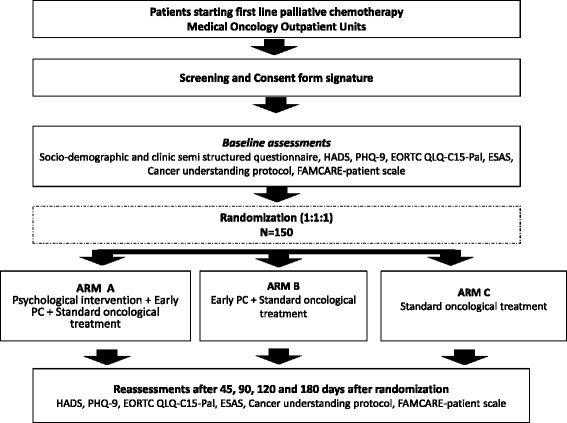
Study flowchart.

This study will be a randomized open-label, phase II clinical trial with two intervention arms and a control group. Figure 1 summarizes the study design.

### Eligibility criteria

#### Inclusion criteria

- Age ≥18 years and <75 years.
- Adequate knowledge about the cancer diagnosis.
- First-line palliative chemotherapy.
- Eastern Cooperative Oncology Group Performance Status (ECOG-PS) ≤2.
- Life expectancy >6 months and <24 months (according to the medical oncologist).
- One of the following diagnoses: metastatic or unresectable recurrent breast cancer, stage IIIC or IV recurrent platinum-resistant ovarian cancer, metastatic or unresectable recurrent cervix cancer, metastatic or unresectable recurrent endometrial cancer, metastatic or unresectable recurrent head and neck cancer (after previous radiotherapy), hormone-refractory metastatic or unresectable recurrent prostate cancer, metastatic or unresectable recurrent genitourinary cancer, metastatic or unresectable recurrent non-small cell lung cancer, extensive-stage or recurrent small cell lung cancer, or metastatic or unresectable recurrent gastrointestinal cancer.

#### Exclusion criteria

Patients can be excluded from the study for any of the following:

1. Any current psychological treatment due to a psychological disorder.
2. Current antidepressant use to treat depressive disorders and/or anxiety.
3. Regular follow-up in the PC department due to advanced cancer or an immediate referral to PC by the assistant physician.
4. Any cognitive deficit or attention problem that could interfere with the ability of patients to answer questionnaires or understand the study aims (according to the investigator).
5. A current or previously established diagnosis of any of the following psychological conditions: substance-related disorders, schizophrenia and other psychotic disorders, mood disorders (depressive disorders, bipolar disorders), anxiety disorders, dissociative disorders, personality disorders, and/or a history of a suicide attempt.
6. Single resected metastasis.
7. Any comorbid condition that would interfere with patient safety, compliance with the study, or the interpretation of the results according to the investigator.

### Data collection and randomization

The psychological intervention will be conducted by psychologists trained for the study. Data collection will be conducted by a team of research coordinators from the Center for Research Support at Barretos Cancer Hospital (BCH; Barretos, SP, Brazil).

A trained member of the Center for Research Support, not involved in data collection or statistical analysis, was assigned to handle the randomization process, using tables of random numbers. The study participants will be randomized (1:1:1) into arms A, B or C. Randomization will be based on blocks of six patients and will be stratified as follows: breast, gynecological, gastrointestinal, urological, head and neck, and thoracic. The study arms will be as follows:

1. Arm A (experimental): psychosocial plus early PC, including five weekly psychosocial interventions based on CBT plus early PC (a first medical consult at the Palliative Care Service will be scheduled for 2 to 3 weeks from the study inclusion date and every 3 to 4 weeks thereafter).
2. Arm B (experimental): early PC (a first medical consult at the Palliative Care Service will be scheduled for 2 to 3 weeks from the study inclusion date and every 3 to 4 weeks thereafter).
3. Arm C (no intervention): standard oncologic care.

### Measures

The Hospital Anxiety and Depression Scale (HADS) is a widely used tool for screening cancer patients for anxiety and depression [13]. The HADS consists of 14 items that are divided into two subscales of seven items each (HADS-A and HADS-D). The score for each item ranges from 0 to 3. A score of 0 is the minimum score for each subscale, and 21 is the maximum score. The psychometric properties of HADS have previously been assessed in the Brazilian population and are considered adequate [14].

The Patient Health Questionnaire (PHQ-9) is derived from the Primary Care Evaluation of Mental Disorders (PRIME-MD) screening questionnaire for depressive symptoms, which was originally developed to screen patients for depression, anxiety, alcohol abuse, somatoform disorders, and eating disorders [15]. The PHQ-9 was validated for use in Brazil in 2013 and is considered a useful tool for screening depression symptoms [16]. The PHQ-9 consists of nine questions that examine the presence of major depressive episode symptoms (Diagnostic and Statistical Manual of Mental Disorders IV, DSM-IV) using the 4-point Likert scale (0 to 3) for a total of 27 points.

The European Organization for Research and Treatment of Cancer Quality of Life Questionnaire Core 15 Pal (EORTC QLQ-C15-Pal) consists of 15 items, and 14 of these items are answered using 4-point Likert-type scales (1 to 4). In addition, a single item assesses overall QL, which is measured using a numerical scale that ranges from 1 to 7. The QLQ-C15-Pal assesses two functional scales (physical and emotional), two symptom scales (fatigue and pain), and five single-item scales (nausea/vomiting, dyspnea, insomnia, loss of appetite, and constipation) [17]. The EORTC-QLQ-C15-Pal was validated for use in Brazil in 2013 with adequate psychometric properties [18].

The Brazilian version of the Edmonton Symptom Assessment Scale (ESAS-br) consists of a visual numeric scale with scores of 0 to 10 to assess ten symptoms, including pain, fatigue, drowsiness, nausea, depression, anxiety, loss of appetite, feeling of wellness, breathlessness, and insomnia [19]. The ESAS-br was associated with adequate psychometric properties when used in a Brazilian population (Paiva *et al*., unpublished data).

The Disease Awareness Protocol is a translated version of the protocol on cancer awareness and treatment that was used by Temel *et al*. and was adapted for this study [6]. This protocol assesses patient perception regarding their chance of a cure and the objective of chemotherapy (see Additional file 1).

The Family Satisfaction with End-of-Life Care (FAMCARE)-Patient scale, originally titled FAMCARE, was developed to assess the satisfaction of family caregivers of advanced cancer patients with healthcare [20]. Subsequently, Lo *et al*. [21] modified the original items to transform this instrument into a 16-item scale for assessing the satisfaction of patients with advanced cancer who undergo outpatient treatment. The most current version of the scale includes 13 items with 5-point Likert-type scale responses that range from ‘very dissatisfied’ (a score of 1) to ‘very satisfied’ (a score of 5) [22]. One item, ‘emotional support provided by the healthcare team’, was added to this questionnaire in the present study because of the nature of the intervention under study. The Portuguese version that was used in the study underwent translation, cultural adaptation, and pre-testing according to Beaton *et al*. [23].

### Interventions

#### Psychosocial intervention based on cognitive behavioral therapy methods

Weekly individual sessions of 40 to 50 minutes in duration will be performed in a properly equipped room for the reception and accommodation of participants. The care protocol that was developed for the present study was based on the method of structuring sessions as proposed by Beck [24] and was adapted from a previous study [25]. The psychosocial intervention is described in detail in Table 1.

**Table 1:** **Detailed structured psychosocial and educational intervention**

**Weekly**	**Planned activities**
1	• Introduction of the therapist/patient and establishment of rapport
	• Objective measurement of depression and anxiety symptoms
	• Psychoeducation: disease - explanation of the cancer diagnosis and current health status; symptoms - a discussion of uncomfortable symptoms secondary to the cancer; adverse events - an explanation of adverse events that result from treatment procedures; PC - What is it? What is it for? For whom is it provided? When is it provided? How is it provided? Where is it provided?
	• Delivery of the ‘Educational Booklet’ that was developed specifically for this study^1^
	• Establishment of therapeutic goals
	• ‘Partnership Agreement’ - the moment when the therapist and the patient sign a commitment to walk together on the path that will be traveled by both during the five proposed sessions
	• Establishment of the dates and times of the sessions
	• Session summary and feedback
2	• Assessment of weekly progress and a bridge to the previous session
	• Objective measurement of depression and anxiety symptoms
	• Reinforcement of the therapeutic goals that were set in the previous session
	• Early PC - description of the benefits of simultaneous monitoring by a PC team for advanced cancer patients who are submitted to palliative chemotherapy and presentation of the explanatory figure ‘Goals of Care’ [35]
	• Delivery of the Coping Card^2^
	• Session summary and feedback
3	• Assessment of weekly progress and a bridge to the previous session
	• Objective measurement of depression and anxiety symptoms
	• Anxiety mechanisms: perceived symptoms and how to monitor these symptoms
	• Relaxing training - image association
	• Session summary and feedback
4	• Assessment of weekly progress and a bridge to the previous session
	• Objective measurement of depression and anxiety symptoms
	• Mechanisms of depressive symptoms: perceived symptoms and how to monitor these symptoms
	• Identification of automatic thoughts (thought > emotion > behavior), explanation of how thoughts affect emotions and therefore behaviors, and elucidating the key role of thought on emotion and behavior
	• Patient preparation for discontinuation following the next session
	• Delivery of the Coping Card1
	• Session summary and feedback
5	• Assessment of weekly progress
	• Objective measurement of depression and anxiety symptoms
	• Retrospective of the methods used in the previous sessions, including progress, difficulties, commitment, and learning
	• Discussion of the patient’s ability to control and understand the process as a way to prevent serious symptoms
	• General feedback

^1^The Educational Booklet contains practical information in layman’s terms about the possible symptoms secondary to chemotherapy treatment and symptoms that are often associated with advanced cancer. This booklet explains the importance of the clinical support that is provided by teams that specialize in palliative care.

^2^The Coping Cards were prepared using stimulating sentences, and guidelines will be provided during the sessions regarding the appropriate moments when the cards should be read or remembered.

#### Early palliative care

The Palliative Care Unit of BCH offers an outpatient clinic and an inpatient ward with 52 beds that are dedicated to cancer patients who are receiving PC. Six PC doctors were asked to participate in this study and have received training. The Palliative Care Unit includes a medical team and a multidisciplinary team of nurses, nurse assistants, nutritionists, physical therapists, psychologists, speech therapists, occupational therapists, music therapists, dentists, and social workers. An evaluation of the research participants by members of the multidisciplinary team may be requested according to the evaluation by the primary physician of the study.

All of the patients will be evaluated by PC physicians every 3 ± 1 weeks, and the first appointment should occur 2 to 3 weeks after randomization. Therefore, the first PC appointment will occur after the first two sessions of the psychosocial and educational intervention for participants who are allocated to Arm A. The appointments will follow a standard care protocol, which shall be duly recorded in medical records using a standardized protocol for filling out the forms.

The participants in Arm C may receive a routine PC evaluation depending on the indication from the attending oncologist. The patients in Arms A and B who have not undergone at least one session of PC and the patients in Arm A who have not participated in the first two sessions of the psychosocial and educational intervention will be excluded from the statistical analyses.

### Primary outcome measures

The primary outcome measures are listed below:

1. Descriptive results about the feasibility of the interventions (the average duration of each intervention session in minutes, the number of absences and the reasons for non-attendance in the intervention groups, the accrual rate, and the reasons for ineligibility).
2. Changes in satisfaction with care from baseline according to the FAMCARE-Patient scale at days 45, 90, 120 and 180.
3. Changes in depression symptoms from baseline according to the HADS-D and PHQ-9 at day 90.

### Secondary outcome measures

The secondary outcome measures are listed below:

1. Changes in depressive symptoms from baseline according to the HADS-D and PHQ-9 at days 45, 120 and 180.
2. Changes in anxiety symptoms from baseline according to the HADS-A at days 45, 90, 120 and 180; the proportion of patients who believe that their cancer is curable as measured using an adapted instrument to evaluate patient awareness (all of the patients will have incurable advanced cancers; therefore, patients who claim that their disease is curable will be interpreted as not being adequately aware of their prognosis).
3. Changes in cancer symptoms from baseline according to the ESAS-br at days 45, 90, 120 and 180; changes in QL from baseline according to the EORTC QLQ-C15-Pal at days 45, 90, 120 and 180.

### Statistical analysis

The demographic and clinical pretreatment characteristics of the patients will be compared between the study arms (A *versus* B *versus* C) using the analysis of variance (ANOVA) test for independent measurements (for the continuous variables) and the chi-squared test (for the categorical variables). Data normality will be assessed using the Shapiro-Wilk test [26] and the data distribution patterns. Cohen’s *d* effect size will be calculated according to the mean difference between the two treatment evaluations divided by the pooled standard deviation of the sample. The analysis of repeated measurements will be performed for each type of outcome at 45, 90, 120 and 180 days after randomization, and these results will be compared to those at the beginning of the study. The effect size will be calculated between Arms A versus C, B versus C, A versus B, and also Arms A plus B versus C. The effect size between the groups will be considered small (0.20 to 0.49), moderate (0.5 to 0.79) or large (≥0.80) [27]. The scores for depression and anxiety symptoms and QL will be compared over time using a mixed model analysis of variance (ANOVA) for repeated measurements. The disease awareness scores and the rate of end-of-life invasive procedures will be compared using the chi-squared test (or Fisher’s exact test). A *P* value <0.05 will be considered statistically significant. The statistical software SPSS v.21 will be used for the statistical analyses.

### Sample size calculation

For the a priori power analysis, GPower software v.3.0 (Heinrich-Heine-Universität, Düsseldorf, Germany) [28] was used to calculate the minimum sample size required for this study. Cohen’s effect size was randomly considered moderate to large (Cohen’s d = 0.65) according to decreased HADS-D and PHQ-9 scores when the experimental arms (A or B) were compared with the control group (Arm C). The estimated sample size would be 39 participants in each arm for a difference between independent means, α of 5% (two-tailed hypothesis) and a power of 80%. Each group shall have a total of 50 patients considering a monitoring loss rate of 25% to 30%. A study of this size will have 80% power to detect differences between Arms A plus B versus Arm C on the depression symptom scores associated with an effect size of 0.5 and alpha of 5%. This study is preliminary in nature, and a key objective of this study is to assess the feasibility and acceptability of a new clinical intervention. Furthermore, this study aims to assess the magnitude of the effect of adding a psychosocial intervention to early PC, which has not been determined according to the literature and would be useful in planning a future definitive phase III trial.

### Monitoring committee

An independent monitoring committee that consists of staff from the Center for Research Support (Coordenadores de Pesquisa do Núcleo de Apoio ao Pesquisador, NAP) of BCH will monitor the collection and analysis of the study data.

### Interim data analysis

An interim analysis is planned for 90 days after the inclusion of 20 participants with complete data in each arm. If Cohen’s effect size between Arms A and B is <0.2 the authors will consider ways to strengthen the psychosocial intervention.

### Ethical aspects

All of the individuals who meet the inclusion criteria will be invited to participate in the study. Each participant will voluntarily sign an informed consent form. The research protocol for this study was developed according to the standards of the National Health Council Resolution number 466/12 and the guidelines of the Declaration of Helsinki. The study protocol was approved by the Research Ethics Committee of the Barretos Cancer Hospital (n° 699/014).

## Discussion

The feasibility and impact of a brief psychosocial and educational strategy, which is based on CBT, will be evaluated in patients with advanced cancer who start palliative chemotherapy to determine whether the patients will be in a better condition when assessed for the first time by a PC team. We believe that the benefits of psychological intervention shall be synergistic to secondary emotional benefits from the early integration of PC. Therefore, this study will evaluate a new strategy for the transition into PC. Furthermore, the inclusion of early PC with or without a brief psychosocial and educational strategy will be compared to standard oncological care in a control arm (first-line palliative chemotherapy) in which patients are referred to PC by the attending physician.

Identifying the reasons patients are referred to PC services is essential because of the benefits of early PC in patients with advanced cancer [6-8,29]. Patients are most likely referred to PC too late even at oncology reference centers [9]. Two randomized phase III trials [6,7] found that early PC was associated with lower levels of depression symptoms compared with standard oncologic treatment. This study aims to confirm this benefit and to examine whether the psychosocial and educational strategy can reduce depression symptoms over time. This original approach may be useful in hospitals with settings similar to those at BCH. This large hospital is dedicated exclusively to oncology and has a well-structured PC service; however, this service is associated with significant patient stigmatization. Patients often perceive the PC unit as being exclusively for dying patients, which complicates their acceptance of early referral to PC.

The attribution of meaning is important for detecting maladaptive behaviors, which are modifiable through interventions according to CBT [30]. CBT is based on the premise that cognition affects behavior; however, cognition may be monitored and changed and the desired behavioral change may be achieved through cognitive change [31]. CBT aims to enable individuals to identify and modify their distorted or dysfunctional automatic thoughts [32]. Evidence suggests that CBT may benefit advanced-stage cancer patients by reducing anxiety symptoms, improving QL [33], and decreasing psychiatric disorders, which have been observed in cancer patients who were classified as high-risk for the development of such disorders [25]. Therefore, the study population in the present study (advanced-stage cancer patients who start palliative chemotherapy) may be considered at a high risk for developing depression. Psychoeducation is another key tool used in CBT. In a previous clinical trial, cancer patients were submitted to a psychoeducational intervention. This strategy was effective in improving disease awareness rates, and the patients were better equipped to deal with problems, had lower levels of isolation and guilt, and felt a sense of belonging to the group. A broad sharing of experiences and lessons by other patients with advanced cancer was observed [34].

The strategy that has been proposed in this study is novel and has not yet been assessed in this context. BCH receives patients from all of the states in Brazil, particularly patients from the Southeast, Midwest and North regions because this hospital is recognized as a reference institution in the Brazilian public health system. Despite being monocentric, this study will assess the impact of the intervention in a culturally heterogeneous population with different needs, and the results of this study may be generalized to the entire Brazilian population.

The study has several limitations and challenges. The main limitation is the small sample size. This study is preliminary and will assess the feasibility of a new intervention and provide initial data on the magnitude of its benefit. Therefore, the statistical significance of this intervention is less important than the main objectives of the study. A larger confirmatory study may be necessary depending on the results of this phase II study. This study will include patients without underlying psychological problems. We anticipate difficulties in enrolling patients because many advanced cancer patients receive psychological support and use antidepressants as part of standard care. Another difficulty in enrolling patients may be the stigma regarding the PC unit and psychological approaches. However, those difficulties will be analyzed in the feasibility assessment.

## Trial status

Patient enrollment in the study began in August 2014. The last patient is expected to be included in January 2017.

## Additional file

Additional file 1:
**Disease awareness protocol.** Description of the disease awareness protocol used in the study.

## Abbreviations

BCH: Barretos Cancer Hospital
CBT: cognitive behavioral therapy
ECOG-PS: Eastern Cooperative Oncology Group performance status
ESAS-Br: Brazilian version of the Edmonton Symptom Assessment System
FAMCARE: Family Satisfaction with End-of-life Care
HADS: Hospital Anxiety and Depression Scale
PC: Palliative Care
PHQ-9: Patient Health Questionnaire
PREPArE trial: Pre-palliative Emotional Care Trial
QL: quality of life
