# Supplementary material for: A phase II study in advanced cancer patients to evaluate the early transition to palliative care (the PREPArE trial): protocol study for a randomized controlled trial
Source: Trials. 2015 Apr 12;16:160. doi: 10.1186/s13063-015-0655-8 (PMC4413544; doi:10.1186/s13063-015-0655-8)
Supplement: Additional file 1: — Disease awareness protocol. Description of the disease awareness protocol used in the study. [file 13063_2015_655_MOESM1_ESM.pdf]

Additional file 1

Disease awareness protocol

The following questions will assess your awareness of the disease that you are being treated for at this hospital. Please be honest and answer what you really think.

1. In your opinion, is your illness (your cancer) curable?

Yes ☐ No ☐

2. What are the goals of your treatment:

You may mark as many answers as you feel appropriate.

a. To help you live longer	Yes ☐	No ☐
b. To make you feel better	Yes ☐	No ☐
c. To completely cure your disease	Yes ☐	No ☐
